# FastqCA: an effective FASTQ compressor through 2D spatial redundancy reduction

**DOI:** 10.3389/fgene.2026.1827471

**Published:** 2026-08-14

**Authors:** Shiyang Long, Qingting Wei, Yanni Zou, Yirui Zhou, Jie Lei, Jiaxu Li

**Affiliations:** 1 School of Software, Nanchang University, Nanchang, Jiangxi, China; 2 School of Mathematics and Computer Sciences, Nanchang University, Nanchang, Jiangxi, China

**Keywords:** cellular automaton, genome assembly, lossy compression, quality score, sequence compression

## Abstract

High-throughput sequencing generates vast amounts of FASTQ data, posing significant storage and transmission challenges. We propose FastqCA, a reference-free, cellular automaton-based compressor designed to exploit two-dimensional spatial redundancy across reads and positions. FastqCA applies a cellular automaton-based predictive-modeling algorithm to the nucleotide sequence stream in both modes, and to the Q4-quantised quality score stream in lossy mode. Evaluation against representative FASTQ compressors, including FQZcomp, DSRC2, fastqz, Scalce, Quip, NAF and SPRING, shows that FastqCA achieves competitive or improved compression ratios on the benchmarked datasets. Genome assembly analysis using QUAST confirms that data compressed by FastqCA maintains assembly quality comparable to the original data across key metrics such as N50 and total length. By effectively utilizing spatial context, FastqCA provides an efficient solution for large-scale sequencing data archival and transmission. FastqCA can be downloaded from https://github.com/XXhaos/FastqCA and freely available for non-commercial usage.

## 1 Introduction

The decreasing cost of high-throughput sequencing (HTS) has led to the gradual popularization of whole genome sequencing. However, along with the rapid growth of data generation, the storage and transmission of sequencing data has gradually become a very significant challenge. It is estimated that the total amount of sequencing data may reach between 2 and 40 exabytes annually, posing substantial challenges for data storage and transmission (D et al., 2015). Such a huge amount of data not only puts severe demands on the storage infrastructure, but also significantly increases the long-term storage costs and complexity of data transmission (Roguski et al., 2018).

FASTQ files, as one of the standard formats for high-throughput sequencing data, have a wide range of applications in storing nucleotide sequences, quality scores, and identifiers. Because of its native redundancy, its effective compression has become an urgent problem to be solved (Bonfield and Mahoney, 2013). Usually the redundancy of a FASTQ file comes from the repeating of reads and their data sections, each data section having its characteristic. The existing general compression techniques, such as Huffman (Huffman, 2007), LZ77 (Ziv and Lempel, 2003), arithmetic coding (Witten et al., 1987), Burrows Wheeler transform compression (Burrows, 1994), PPM (Cleary and Witten, 2003), etc., only compress FASTQ sequencing data as plain text without considering its unique characteristics, resulting in unsatisfactory compression results. To address this issue, researchers have proposed a variety of FASTQ-specific compression methods, which can be broadly categorized into reference-based and reference-free compression (Bonfield and Mahoney, 2013; Zhang et al., 2015).

Reference-based compression methods such as Fastqz (Bonfield and Mahoney, 2013) and CRAM (Fritz et al., 2011), utilize existing reference genomes to compress sequencing data, significantly reducing data storage requirements by storing only the portion of the nucleotide sequences that differs from the reference sequence, e.g., mutations, insertions, or deletion sites. The core steps of such approaches involve mapping the *de novo* sequencing data to the reference sequence and recording the differences between the two to enable efficient compression. In addition, GReEn (Pinho et al., 2012) further enhances the compression effect through arithmetic coding (Witten et al., 1987), which is particularly suitable for high similarity genomic data. However, these methods rely on high-quality and complete reference sequences, and the compression effect decreases significantly when dealing with genomes with high variability or lacking reference sequences, e.g., metagenomics where a clear reference genome is not available (Jones et al., 2012). The dependence on reference sequences and inadaptability on highly variable genomes limit the wide application of reference-based compression methods.

In contrast, reference-free compression methods provide more flexible and efficient solutions, especially for genomic data with high diversity and imperfect reference sequences. This type of methods achieve compression by mining redundancy in the FASTQ file itself. For example, Quip employs *de-novo* assembly, which reduces storage requirements by assembling a large number of reads to discover overlaps between nucleotide sequences (Jones et al., 2012). Nucleotide Archival Format (NAF) provides lossless compression for FASTA/FASTQ files with fast compression, making it efficient for storage and distribution of genomic data (Kryukov et al., 2019). In addition, DSRC2, an industry-oriented compression tool that strikes a good balance between compression efficiency and processing speed, is widely used in large-scale genome projects (Roguski and Deorowicz, 2014). However, some of the reference-free compression methods gather similar nucleotide sequences by changing the order of reads. For order-insensitive workloads (e.g., k-mer counting, error correction/normalization, and *de novo* assembly graph construction) or offline archiving where a stored permutation is restored at decode time, read reordering can further improve compression at the cost of additional memory and reduced streamability. It seems not to exert influence on genome assembly, quality control and other downstream analysis, whereas reordering of reads may cause the mapping of paired-end sequencing data failed. Moreover, the non-reference based compression methods often place their attention on the compression of nucleotide sequences, few of them employing special strategies on compression of quality scores in FASTQ files.

Quality scores account for a high proportion of FASTQ files, and how to compress quality scores efficiently has gradually become a research focus. The quality scores are used to assess the reliability of each base obtained by sequencing. Unlike nucleotide sequences, the quality scores usually show a high degree of randomness and low redundancy and so it also becomes the most difficult part of the whole file to compress. To cope with this problem, researchers have proposed a variety of compression algorithms specialized for quality scores. For example, SCALCE employs a lossy compression strategy (Hach et al., 2012), which achieves high compression ratios by a lossy transformation of the quality values of base pairs. However, it is only applicable to cases where the quality score accuracy is not required but a significant reduction in storage space is needed, such as large-scale genome sequencing projects. The AQUa framework is able to adaptively select the most suitable coding tools and strategies for lossless compression based on the characteristics of quality scores (Paridaens et al., 2018; Chen et al., 2022). This means that during the compression process, AQUa dynamically analyzes the data and then decides which coding method to use, such as Differential Function Coder (DFC), Convolutional Predictor (CVP) (Paridaens et al., 2018; Cho and No, 2021), etc., in order to achieve the best compression results. Although lossy compression has a significant advantage in compression ratio, it is still controversial in practical applications, especially in medical and clinical fields that require high data integrity, which is why AQUa chooses to focus on lossless compression strategies.

Hybrid schemes are effective data compression methods that employ a combination of lossy and lossless techniques. These approaches leverage the strengths of both methods, ensuring that critical information is preserved while achieving efficient compression. In practice, these approaches typically implement lossless compression on fields that are essential for exact reconstruction, and controlled lossy transformations on components that are less informative. This process substantially reduces storage requirements. SPRING operationalises this paradigm in a reference-free setting by retaining nucleotide sequences and read pairing exactly, while applying calibrated binning to quality scores and relaxing constraints on read identifiers and global order. This approach has been shown to yield substantial space savings while exerting minimal influence on subsequent analyses (Chandak et al., 2019). Fastqz employs a three-stream design that treats identifiers, sequences, and quality strings separately, combining preprocessing and context modelling. When a reference is supplied, the system replaces long matches with compact pointers and a few mismatch positions to permit exact reconstruction given the same reference (Bonfield and Mahoney, 2013). In this work, the three-stream decomposition is adopted, with identifiers, sequences and quality strings processed independently. This enables channel-specific transforms, while ensuring that exact reconstruction is preserved for critical fields and that calibrated binning is applied to quality values where appropriate.

In this paper, we propose FastqCA, a reference-free, cellular-automaton–based compressor for FASTQ files that reduces storage by exploiting two-dimensional spatial redundancy across reads and positions while preserving the original read order.

The idea of exploiting redundancy across multiple reads instead of coding each read in isolation has been explored by several modern FASTQ and genomic compressors. FaStore groups reads according to sequence similarity before compression and supports both lossless and lossy reference-free FASTQ compression (Roguski et al., 2018). SPRING brings similar reads together by hash-based read reordering and then applies specialized component-wise compression to identifiers, nucleotide sequences and quality scores in a reference-free setting (Chandak et al., 2019). FQSqueezer is a k-mer-based compressor that follows the prediction by partial matching (PPM) and dynamic Markov coding ideas for sequencing data compression (Deorowicz, 2020). Genozip provides a universal genomic compression framework that supports FASTQ together with SAM/BAM/CRAM, VCF, GVF, FASTA, PHYLIP and 23andMe formats, and can optionally use a reference genome for sequence-data compression (Lan et al., 2021). These methods show that FASTQ and genomic compression remain active research areas. FastqCA takes a different view of FASTQ data: reads in a chunk are organized into an integer mapping matrix with two dimensions, read and position, and the value at every position is predicted from its above, above-left and left neighbours by a cellular automaton whose rule table is dynamically updated during scanning. The novelty of this work therefore lies in the two-dimensional cellular-automaton formulation, rather than in claiming that cross-read redundancy exploitation itself is new.

Preserving read order enables strictly streaming operations, stable archival checksums, and one-pass lane/tile quality control using header metadata. Therefore, FastqCA is specifically designed for high-density archival and bandwidth-limited transmission pipelines, where minimizing storage footprint is prioritized over processing throughput. FastqCA adopts a hybrid, stream-wise strategy that nucleotide sequences are compressed losslessly, whereas quality scores are encoded either losslessly or via a controlled four-level (Q4) quantization, depending on the chosen mode. The main contributions of this paper are as follows:

- A partitioning compression framework is presented where sequencing data is split into fixed-size chunks and each chunk is partitioned into the streams of IDs, nucleotide sequences and quality score rows. Separate processing approaches are applied to different data streams to fully utilize their respective characteristics for efficient compression.
- A two-dimensional predictive-modeling encoding algorithm based on a cellular automaton is proposed. Different from the existing reference-free compressors exploiting cross-read redundancy, FastqCA organizes the reads in a chunk into a two-dimensional integer mapping matrix, and predicts each value from its above, above-left and left neighbours through a dynamically updated rule table. The data stream is then encoded as a sparse matrix of the difference between the original and predicted values.
- The lossless and lossy compression modes are both available in compressing quality scores. Lossless compression ensures complete data recovery in decompression, while lossy compression significantly reduces the amount of quality scores through the Q4 quantization with minimal impact on downstream biological analysis.

The rest of this paper is organized as follows. Section 2 presents an overview of the proposed FastqCA compression method and describes its implementation detail. Section 3 presents the experimental evaluation on compression performance and assembly quality. Finally, Section 4 concludes the paper and highlights some future work directions.

## 2 Materials and methods

### 2.1 Overview of FastqCA

**FIGURE 1:**
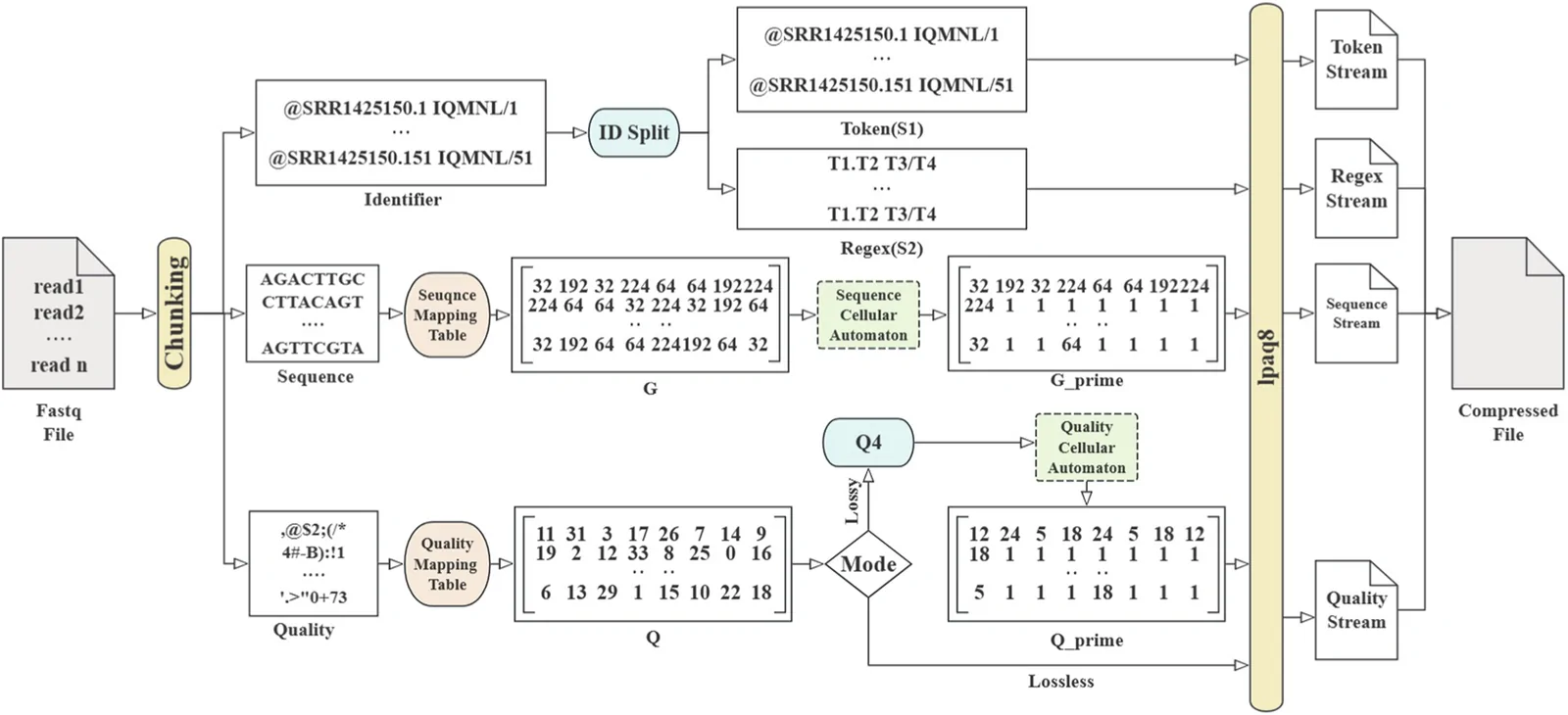
Overview of the FastqCA compression method.

The compression procedure of the proposed FastqCA compression method is illustrated in Figure 1. Firstly, FastqCA partitions the chunks of a FASTQ file into three types of streams, i.e., IDs, nucleotide sequences and quality scores. Before compressing the data streams, FastqCA preprocesses the streams and converts them to more compressible format. For the ID stream, FastqCA uses semantic analysis techniques to understand the meaning of tokens then separates the tokens into data fields and their structures in regular expression. For the nucleotide sequence stream, FastqCA transforms the sequences to an integer matrix G , constructs a cellular automaton to model and predicts the G matrix. Then the G matrix is converted to the matrix $G_{prime}$ of the differences between the original data and the predicted data. For the quality score stream, FastqCA transforms score values to an integer matrix Q , and then preprocess Q according to working in the lossless or lossy compression modes. In the lossless mode, the Q matrix is forwarded to the back-end compressor directly. The reason for this design, a trade-off between rule-table size, per-cell scan cost and prediction hit rate at $|\Sigma_{Q}|=94$ , is detailed in Section 2.5. In the lossy mode, the quality scores of Q will be quantized into four levels (called Q4 quantization) and encoded by the cellular automaton like doing with the nucleotide sequence stream. Since the Sanger or Solexa quality scores (Cock et al., 2010) often include too many grades of score values, the Q4 quantization we adopted has very little effect on assembly polishing of the sequencing data. Finally, the preprocessed results are compressed by a common data compressor on the back end and written in the output file. The common data compressor used in FastqCA is LPAQ8^1^, which adopts a context mixing compression algorithm and the parallel model processing. Concretely, LPAQ8 uses multiple compression models that can operate simultaneously in order to better capture the redundancy of the data. Different models may be suitable for different types of data. One disadvantage of LPAQ8 is that it can not handle files with a size exceeding 1G bytes. So we choose LPAQ8 for further compression of the data streams after preprocessing. In this paper, we set the default parameter, option ‘9’, which gives the best compression results.

### 2.2 Partitioning

The FASTQ file contains a number of reads, each of which consists of four components: ID, nucleotide sequence, the ‘+’ symbol and quality scores. As shown in Figure 1, ID is the first row of a read starting with the ‘@’ symbol and usually used to record metadata such as sequencing platform information, sequence length, etc. The nucleotide sequence in the second row is nucleotide bases generated by the sequencer. This is typically composed of a series of ‘A’, ‘T’, ‘C’, ‘G’ and ‘N’ characters. The third row starts with a ‘+’ symbol, which acts as a placeholder and does not contain any valid information, so it is not required to be saved in compression. The quality score row contains the score values representing the sequencing quality of each nucleotide in the sequence, formatted as ASCII characters.

Since the IDs, nucleotide sequences and quality scores in the reads have different characteristics, we split the reads into chunks of 128 MiB (134,217,728 bytes) by default and then partition them into streams of IDs, nucleotide sequences and quality scores. Our idea of partitioning compression is to extract the characteristics of different types of data streams and compress them separately before feeding them to the back-end compressor.

The default chunk size of 128 MiB is determined by an engineering trade-off. Larger chunks expose more local redundancy to the front-end CA model, but they also increase the number of FASTQ records, integer matrices, image buffers and temporary streams held or generated per active thread before LPAQ8 coding. In addition, the LPAQ family uses substantial memory at high compression options; for example, the public LPAQ documentation reports that option 9 uses about 1542 MB. Therefore, chunk size directly affects the balance among compression ratio, peak memory and compression speed. To quantify this trade-off, we evaluated FastqCA at chunk sizes of 16 MiB, 32 MiB, 64 MiB and 128 MiB on the same ten datasets in lossless mode with--threads 4, with the per-dataset values reported in Supplementary Table S1. Across the ten datasets, the compression ratio increases monotonically with the chunk size; the peak memory increases approximately monotonically with the chunk size; and the compression speed shows a weaker dependence on the chunk size, with the 128 MiB setting reaching the highest throughput on most datasets, likely because the per-chunk LPAQ8 invocation overhead is amortised over more bytes. Therefore, 128 MiB is the default value we adopted on the experimental platform reported in Section 3. It is also exposed as a command-line argument (--block_size), whose default value is 134,217,728 bytes, so that users may adjust it according to their own hardware.

Thread parallelism in FastqCA is applied at the chunk granularity. Chunks produced by the partitioning step are dispatched to a pool of worker processes, and each worker independently runs the full pipeline of one chunk, including identifier splitting, integer mapping, predictive-modeling encoding and the back-end LPAQ8 invocation on each of the four streams. Within a single chunk the four streams are compressed sequentially, and the cellular automaton prediction itself is sequential along the scanning order, since the value at each cell depends on its already-processed above, above-left and left neighbours. The number of worker processes is exposed as the command-line argument --threads, and is set to four for all results reported in Section 3.

For synchronized paired-end FASTQ files, FastqCA does not implement a dedicated joint coding scheme between the two mate files. The mate FASTQ files of a paired-end run are each supplied as an independent input, and compressed separately under exactly the same pipeline described above. Since FastqCA preserves the original read order within every file, the mate-pair correspondence is maintained by positional alignment alone, and no auxiliary pairing index is required.

### 2.3 Identifier compression

The IDs section is the neatest part of reads, as it is generated very systematically by each sequencing instrument. So we can exploit the characteristics of the IDs section to compress it. For example, an identifier may contain the fields of instrument’s name, run identifier, flow cell identifier, ‘x’-coordinate of the cluster within the tile and ‘y’-coordinate of the cluster within the tile. And an identifier starts with ‘@’ followed by the name of the dataset in most cases. Those characteristics of IDs cause the data redundancy of reads in the same FASTQ file, which increases the size of the IDs section.

Usually three types of data fields occur in the identifier section, i.e., the fields that remain constant throughout the file, the fields that have the same value in a set of consecutive reads, and the fields having incremental or decremental values in consecutive reads. So based on these characteristics of the IDs section, our algorithm first scans the IDs section for separators. Then we split the identifiers into data fields by the common separators like dot, colon, underline, space, equal sign, slash and hyphen. After the algorithm finishes scanning the stream of IDs, it generates two new sub-streams. The items in the first sub-stream are composed of the data fields, which are truncated by the separator space. The second sub-stream consists of strings of the separators with the placeholders for the data fields, which represent structure information of the data fields in the IDs and help us to recover the original file during the decompression process.

As shown in Figure 1, the identifier “@SRR14251500.1 IQMWFNS04EUNNL/1” in the stream of IDs will be split by the separators into the four data fields: “@SRR142515001”, “1”, “IQMWFNS04EUNNL” and “1”. The four data fields truncated by the separator space constitute a new item in the sub-stream S1, and the separators as well as the placeholders make up a new item in the sub-stream S2 as follows:

S1: @SRR14251500 1 IQMWFNS04EUNNL 1
S2: T1.T2 T3/T4

After generating the two sub-streams of IDs, we send them to the LPAQ8 compressor for compression and integration.

### 2.4 Nucleotide sequence compression

#### 2.4.1 Data conversion

The nucleotide sequence section of a FASTQ file often consists of five bases ‘A’, ‘C’, ‘G’, ‘T’ and ‘N’. There may be homology, conserved region, base pairing, secondary structure and other complex biological structures across different reads. Therefore we exploit the spatial relation of nucleotide sequences among reads for better compression. We first load the nucleotide sequence stream in every chunk of a FASTQ file into memory and map the bases to integer values according to the rule: the base A to 32, T to 64, G to 192, C to 224, and N to 0. These specific byte values serve as a one-to-one mapping of the five-symbol nucleotide alphabet. Although A, T, G, C, N could theoretically be encoded using 3 bits per symbol, the back-end LPAQ8 entropy coder operates on full bytes. Each nucleotide is therefore represented as a distinct byte (0–255). This mapping not only enables partial bit-level error detection due to differences in the high 3 bits (partial Hamming distance protection), but also ensures byte alignment for efficient network transmission and stream processing. Then the nucleotide sequence stream in a chunk is converted to a matrix G consisting of the five integer values 32, 64, 192, 224 and 0.

#### 2.4.2 Predictive-modeling encoding

**FIGURE 2:**
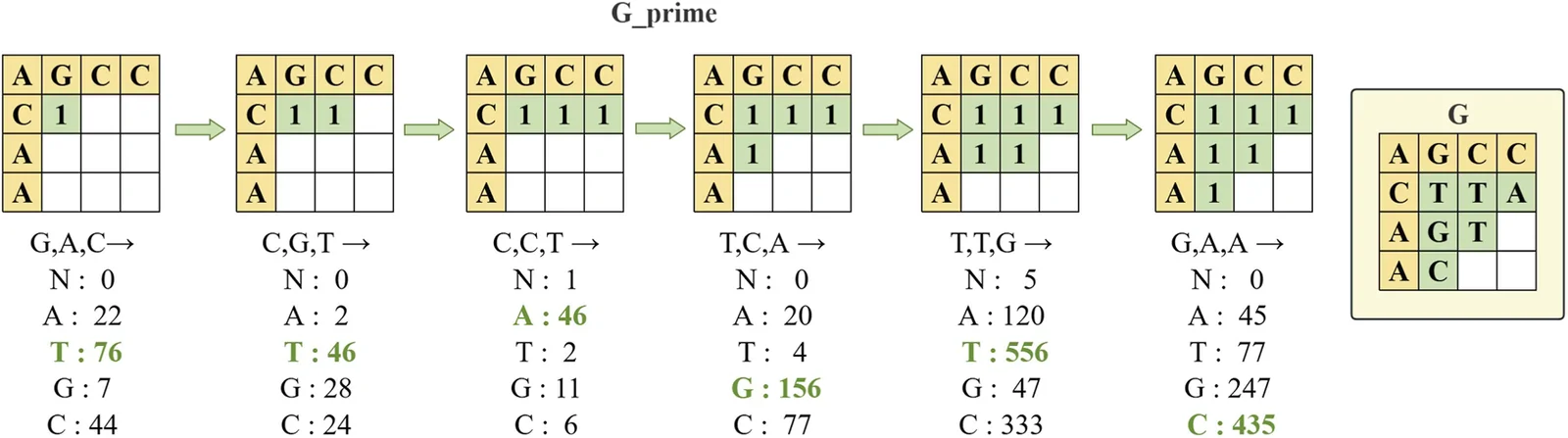
The predictive-modeling encoding procedure for nucleotide sequences in FastqCA.

The core step of our algorithm is predictive modeling the occurrences of integers in the matrix G using cellular automaton. We construct a $G_{prime}$ matrix of the same size as the original matrix G , where all elements have an initial value of 0. The $G_{prime}$ matrix is used to capture the predicted integer value of each nucleotide by cellular automata based on spatial context, which is guided by dynamic local rules in cellular automata. Specifically, the prediction process relies on the values of the neighbors (i.e., above, above left, and left) of the nucleotide at the current position. We determine the most likely integer value for the current position by a set of rules stored in a dictionary called rule_table , which is dynamically updated based on the values observed in the data. The predictive-modeling encoding procedure is illustrated in Figure 2.

The rule_table is a four-dimensional array and the array elements are denoted as rule_table[up,left_up,left,center] . The first three dimensions (denoted as up , left_up and left ) represent the above, above left and left neighbours in the matrix, and the last dimension (denoted as center ) is the value of possible nucleotide occurring at the current position. Each array element corresponds a prediction rule and the value of the array element save the frequency of the rule being used to predict. All array elements have initial values of 0.

At the beginning of the modeling procedure, the algorithm traverses the matrix G from left to right line by line. For every matrix element, the algorithm scans its neighbor elements that locate above, above left and left. Then we query rule_table for the rules where the first three dimensions match the values of up , left_up and left , and compare the values of the array elements including: rule_table[up,left_up,left,32] , rule_table[up,left_up,left,64] , rule_table[up,left_up,left,192] , rule_table[up,left_up,left,224] , and rule_table[up,left_up,left,0] .

Since the values of the five array elements indicate the frequency of the rules being used, the rule with the maximum frequency is selected where the value at the center dimension is the predicted value of nucleotide at the current position. We compare the predicted value with the actual value at the current position in the matrix G . If the predicted value is equal to the actual value, we mark the current position of $G_{prime}$ as 1, indicating a successful prediction; if it is not equal, we set the value of the current position of $G_{prime}$ to the actual value, indicating a failed prediction.

\For example, in Figure 2, after obtaining the original matrix G , a matrix $G_{\mathrm{prime}}$ of the same size is constructed, retaining only the first row and the first column. Prediction proceeds element by element from the upper-left corner. When reading this block, suppose that for the first predicted position, the values of *up*, *left-up*, and *left* are G, A, and C, respectively. In the rule table, the corresponding frequencies for this rule are N :0, A :22, T :76, G :7, and C :44. The most frequent value T is selected as the predicted symbol. By comparing with the original matrix G , if the prediction is correct, it is marked as 1.

The cellular automaton keeps the rule table dynamically. Regardless of whether the prediction is successful or not, we update the frequency of rules in the rule_table . For example, the value of the element rule_table[up,left_up,left,center] is increased by 1 when the corresponding rule is used again.

With this cellular automata based algorithm, we are able to utilize the spatial context of nucleotide to make local predictions and encode the original matrix G as a $G_{prime}$ matrix. After the predictive encoding, the integer ‘1’ will appear at many positions of the $G_{prime}$ matrix, which helps to improve the back-end compression.

To avoid excessive memory usage and to accommodate the input-size limit of LPAQ8, the predictive-modeling encoder loads the reads of one chunk into memory at a time, and different G and $G_{prime}$ matrices are extracted from different chunks. The rule_table is initialized at the beginning of each chunk and is updated only from the cells within that chunk, so the prediction operates on the local statistics of the current chunk.

After the predictive-modeling encoding, we feed the $G_{prime}$ matrix into the LPAQ8 compressor for compression and integration, which achieves high compression rates with the help of the spatial dependencies captured in the predictive modeling procedure. During compression of the whole FASTQ file, LPAQ8 is working under the same setting of default parameters.

In addition, it is worth emphasizing that the rule_table in decompression follows the same pattern of changing used during compression, so we do not need to keep the complete rule_table in the compressed file. By constructing the same rule_table and its rules, the original matrix G can be perfectly recovered during decompression. Therefore, the whole predictive-modeling encoding process is actually the procedure of converting the G matrix into the $G_{prime}$ matrix. The algorithm of predictive-modeling encoding based on cellular automata is shown in Algorithm 1 as follows:

Algorithm 1 **Algorithm of predictive-modeling encoding based on cellular automaton.**

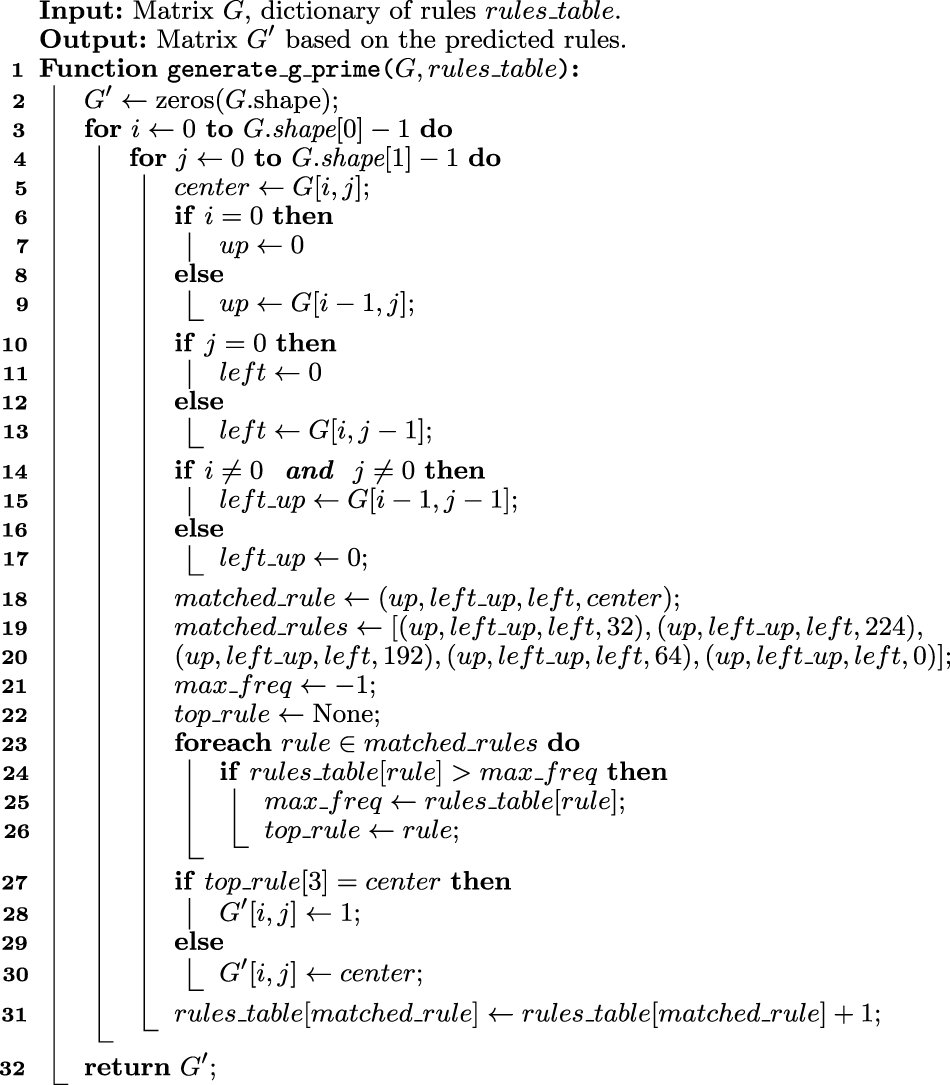

### 2.5 Quality scores compression

#### 2.5.1 Lossless mode

FastqCA provides two encoding paths for the quality score stream. In the lossless path, the symbols of quality scores are mapped to their corresponding ASCII values between 33 and 126, which constitutes a matrix Q similar to the matrix G for nucleotide sequences. The predictive-modeling encoding in Section 2.4 can in principle be extended to the matrix Q . However, we do not apply the cellular automaton on the matrix Q in the lossless mode. The reason is that the alphabet size |Σ| of the matrix Q is much larger than that of the matrix G , which causes unfavourable cost in three aspects, i.e., time, space and compression ratio.

##### 2.5.1.1 Time cost

According to Algorithm 1, the encoding of a chunk of R reads with read length L scans the |Σ| candidate rules to select the one with the highest frequency for each of the R⋅L cells. So the encoding time per chunk is Θ(R⋅L⋅|Σ|) . The three alphabet sizes used by FastqCA are

$$\begin{array}{cccc} \left|\Sigma_{G}\right| & =5 & & \left(\text{A/C/G/T/N}\right),\\ \left|\Sigma_{Q4}\right| & =4 & & \left(\text{four quantised levels}\right),\\ \left|\Sigma_{Q}\right| & =94 & & \left(\text{Phred }33\text{–}126\right). \end{array}$$

Therefore the per-cell encoding of the matrix Q would be about 94/5≈18.8 times slower than that of the matrix G , and about 94/4≈23.5 times slower than that of the Q4-quantised matrix $Q^{\prime }$ used in the lossy mode.

##### 2.5.1.2 Space cost

The rule_table is indexed by a 4-tuple (up,left_up,left,center) , and its worst-case footprint is $|\Sigma {|}^{4}$ . The three alphabet sizes give

$$|\Sigma_{G}{|}^{4}=625,\;|\Sigma_{Q4}{|}^{4}=256,\;|\Sigma_{Q}{|}^{4}\approx 7.81\;\times \;10^{7}.$$

Even with a sparse hash-based implementation in which only observed tuples are materialised, the active key set on the matrix Q in the lossless path is several orders of magnitude larger than that on the matrix G or the matrix $Q^{\prime }$ , which raises pressure on cache locality and per-thread memory.

##### 2.5.1.3 Compression-ratio cost

The hit rate of the predictor is upper-bounded by the dominant conditional probability $\max_{k}P\left(k|C\right)$ . When |Σ| increases from 4–5 to 94, the dominant probability shrinks rapidly under any non-degenerate empirical distribution. So the residual matrix becomes dense and the entropy gain over directly feeding the matrix Q to LPAQ8 collapses. Combining the three costs above, applying the CA-augmented path to the lossless matrix Q would inflate the per-cell scan cost by about 19× and the rule-table footprint by about $10^{5}\times$ relative to the matrix G , while the compression-ratio gain shrinks to a negligible level. This is a trade-off in all three dimensions simultaneously.

##### 2.5.1.4 Resulting design

Therefore, in the lossless path, FastqCA forwards the matrix Q directly to the back-end LPAQ8 compressor. LPAQ8 is a byte-level context-mixing entropy coder, which can exploit the sequential byte-level context that is well-suited to the locally regular structure of quality streams within each read. The CA-based predictive modeling is applied to the matrix G of nucleotide sequences in both modes, as well as to the Q4-quantised matrix $Q^{\prime }$ in the lossy mode, where $|\Sigma_{Q4}|=4$ keeps the above analysis well within the regime in which the predictive modeling is effective. So in the lossless path, the CA-based 2D spatial prediction applied on the matrix G is what differentiates FastqCA from feeding the same chunked matrices through LPAQ8 alone.

#### 2.5.2 Lossy mode

For lossy compression, we use the Q4 quantizer (Rivara-Espasandín et al., 2022) for mapping quality scores to the values with few grades. Although the Q4 quantization process loses a part of the precision of quality scores’ grades, there is no significant effect on assembly polishing and variant calling of contigs and even sometimes the results are better than original data.

The four-level granularity of our quantizer follows the Q4 setting used in previous quality-score compression work, and is further supported by downstream evaluations of reduced-resolution quality scores. Yu et al. (2015) reported improved SNP-calling accuracy after discarding the majority of quality information through the Quartz algorithm. Ochoa et al. (2017) systematically evaluated several lossy quality compression schemes, and reported that the variant-calling performance under some configurations is superior to that obtained from uncompressed data. Most directly relevant to our setting, Rivara-Espasandín et al. (2022) applied a four-level quality quantizer to nanopore sequencing data on a mock microbial community, and observed that the *de novo* assembly polishing produced fewer mismatches per 100 kbp than the non-quantised data, with comparable polishing quality on human data. The bin boundaries we adopt, i.e., Q≤7 , 7<Q≤13 , 13<Q≤19 and Q≥19 , are derived from the Phred relation $P=10^{-Q/10}$ , and correspond to per-base accuracy ranges of approximately <80% , <95% , <99% and ≳99% respectively.

Concretely, we map the quality values less than or equal to 7 to 5, values greater than 7 and less than or equal to 13 to 12, values greater than 13 and less than or equal to 19 to 18, and values greater than or equal to 19 to 24. Each value is taken as the mid-point of its bin, and the top bin is represented by 24 as a typical high-confidence value with per-base error rate about $4\;\times \;10^{-3}$ . Then the Q4 quantizer transforms the Q matrix to the $Q^{\prime }$ matrix with only four possible values, which is a very good fit for our predictive-modeling encoding. We call the predictive-modeling encoding algorithm in Algorithm 1 to encode the quantized Q matrix like doing with the G matrix of nucleotide sequences. The rule_table is still a four-dimensional array, but the array elements have four possible values. After predictive-modeling encoding, the $Q_{prime}$ matrix is obtained. We next feed the $Q_{prime}$ matrix into the LPAQ8 compressor to compress and integrate the quality score stream. This lossy compression method has less impact on biological experiments and can significantly improve the compression rate. Both lossless and lossy compression modes can be selected according to the actual needs.

### 2.6 Decompression

For decompression of the ID section, we first use the decompression algorithm of LPAQ8 to recover the two sub-streams, one of which records the data fields of IDs, and the other of which saves the structure information of IDs. By using these two sub-streams we can restore the complete ID rows in the original reads.

For lossless quality score decompression, our decompression process simply calls the LPAQ8 decompression algorithm to get the Q matrix, and then maps to the corresponding ASCII code based on the values in the matrix. In this way we can restore the quality score section of the original FASTQ file.

For nucleotide sequences and lossy quality scores, the decompression method is similar. First, we get the matrix $G_{prime}$ or $Q_{prime}$ by the decompression algorithm of LPAQ8. Then we use the predictive-modeling method employed in compression to decode the G or Q matrix. Similarly, we define the rule_table and update the frequencies of rules according the neighbours of each matrix element. It is important to note that we only need to process the elements with value ‘1’ in the $G_{prime}$ or $Q_{prime}$ matrices. We start scanning from the top left corner of the matrix $G_{prime}$ or $Q_{prime}$ and update the frequencies of rules that appear in the rule_table one by one. When a value of ‘1’ is scanned in, we read the values of all the array elements rule_table[up,left_up,left,center] where up , left_up and left match the neighbours’ values of the matrix element at the current position. We select the rule with the maximum array element value, and decode the ‘1’ at the current position in $G_{prime}$ or $Q_{prime}$ as the center value of the selected rule, which is actually the predicted data in our algorithm. With this inverse operation, we obtain the original matrix G and Q respectively for decompression of nucleotide sequences and lossy quality scores. Finally, based on the mapping dictionary of G and the ASCII mapping of Q , we can restore the nucleotide sequence section of the original FASTQ file as well as the quality score section after quantization.

## 3 Experimental evaluation

To assess the performance of FastqCA, we conducted a representative comparative evaluation using ten diverse FASTQ datasets. We compared FastqCA with the general-purpose gzip baseline and a set of established FASTQ compressors, including FQZcomp (Bonfield and Mahoney, 2013), DSRC2 (Roguski and Deorowicz, 2014), fastqz (Bonfield and Mahoney, 2013), Scalce (Hach et al., 2012), Quip (Jones et al., 2012), NAF (Kryukov et al., 2019) and SPRING (Chandak et al., 2019). All benchmark runs were performed on FASTQ input without an external reference, and lossless or quality-score lossy settings were evaluated where supported by the corresponding tool. The selected tools provide representative coverage of established general-purpose and FASTQ-specific compression approaches. The evaluation compares compression ratio, speed and memory usage across the applicable benchmark tools, supplemented by downstream assembly evaluation for the lossy compressors included in that experiment. The empirical conclusions in this study refer to the tools, modes and datasets evaluated here.

The experimental scope covers compression and decompression efficiency, ablation studies on the predictive modeling, and downstream genome assembly quality. All tools were executed on a standardized platform (Intel Core i9, 64 GB RAM). For each benchmark compressor, we configured the parameters to prioritize compression ratio over runtime speed (e.g., gzip at −9). The tool-specific command modes and versions are detailed in Supplementary Table S2. For FastqCA, compression and decompression speeds were measured over three separate wall-clock runs per dataset and mode under the benchmark settings described above and the standard deviations are provided in Section 3.3. For the benchmarked tools, the timing measurements were performed as single runs, as the comparison was conducted at an earlier stage of this study.

### 3.1 Datasets

The experimental datasets were selected to ensure a comprehensive evaluation across diverse genomic features and sequencing technologies, as summarized in Table 1. Our collection comprises ten core FASTQ files representing various research domains. We included SRR554369 from the MPEG-G/MPEG HTS benchmark collection used in previous FASTQ compression studies, including HARC (Chandak et al., 2018) and SPRING (Chandak et al., 2019). This accession provides overlap with prior compressor evaluations while the remaining datasets broaden the range of file sizes and sequencing contexts. To validate FastqCA’s performance on larger scales and diverse libraries, we incorporated human genomic data (SRR1210085_1), environmental metagenomes (DRR057887_1), and transgenic monitoring datasets including their respective paired-end reads (SRR14139158 and SRR14626645). Furthermore, complementary library strategies for B. thuringiensis HS18-1 were utilized, featuring both short-insert (SRR2093871_1) and large-insert mate-pair (SRR2093872_1) reads. This selection encompasses file sizes ranging from several hundred megabytes to over 15 GB, enabling a robust evaluation of the algorithm’s scalability and efficiency.

**TABLE 1:** Technical specifications of the ten core experimental datasets.

Accession	Organism/Project context	Technology	Size (bytes)
SRR1210085_1	*Homo sapiens* (genomic DNA)	Illumina HiSeq 2000	15126475212
SRR14139158_1	Transgenic monitoring (*S. lycopersicum*)	Illumina HiSeq 4000	10859255381
SRR14139158_2	Transgenic monitoring (*S. lycopersicum*)	Illumina HiSeq 4000	10859255381
SRR14626645_1	Environmental monitoring	Illumina HiSeq 4000	8845364612
SRR14626645_2	Environmental monitoring	Illumina HiSeq 4000	8845364612
SRR29245815	*B. subtilis* GL-4 (bamboo rat gut)	Illumina HiSeq 4000	3649758240
SRR2093872_1	*B. thuringiensis* HS18-1 (mate-pair)	Illumina HiSeq 2000	1907047029
SRR2093871_1	*B. thuringiensis* HS18-1 (PE library)	Illumina HiSeq 2000	1817852165
SRR554369	*P. aeruginosa* (MPEG-G/MPEG HTS benchmark)	Illumina GA II	788017922
DRR057887_1	Metagenome (environmental sample)	DDBJ/Illumina	570579485

### 3.2 Evaluation metrics

In this study, we adopt a multidimensional evaluation system to comprehensively analyze compression tools in terms of both technical efficacy and biological fidelity, in order to balance the cost of data storage with the reliability needs of downstream bioinformatics analysis. The evaluation metrics are defined as follows:

#### 3.2.1 Performance metrics

We evaluate each compressor on three dimensions: compression ratio, compression speed, and peak memory usage. Let $S_{orig}$ and $S_{comp}$ be the file sizes (bytes) of the original and compressed data; $T_{comp}$ is the elapsed compression time (seconds); $T_{decomp}$ is the elapsed decompression time (seconds).

##### 3.2.1.1 Compression ratio (CR) and weighted average compression ratio (WACR)

CR is the dimensionless ratio of original size to compressed size as shown in Equation 1:

$$CR=\frac{S_{\text{orig}}}{S_{\text{comp}}}.$$ (1)

A larger CR indicates that more storage space is saved. For example, a CR of 12.5 means the compressed file is 12.5 times smaller than the raw FASTQ input, occupying only 8% of the original storage footprint. This metric quantifies how effectively each method reduces the volume of sequencing data that must be stored on an individual dataset.

In addition to the per-dataset CR, we also report the weighted average compression ratio (WACR) over the benchmark corpuscalculated as Equation 2:

$$WACR=\frac{\overset{n}{\underset{i=1}{\sum }}S_{\text{orig},i}}{\overset{n}{\underset{i=1}{\sum }}S_{\text{comp},i}}.$$ (2)

Unlike the arithmetic mean of per-dataset CR values, WACR weights each dataset according to its original byte size and therefore reflects the compression ratio achieved when the complete benchmark corpus is treated as one database.

##### 3.2.1.2 Compression/decompression speed (CS/DS)

Throughput is reported in *megabytes per second* as shown in Equation 3:

$$CS=\frac{S_{\text{orig}}/10^{6}}{T_{\text{comp}}},\;DS=\frac{S_{\text{orig}}/10^{6}}{T_{\text{decomp}}}\;\left(\text{MB/s}\right).$$ (3)

here, $T_{comp}$ and $T_{decomp}$ denote the elapsed wall-clock time for compression and decompression, respectively. Higher values indicate faster processing. For example, a CS of 200 MB/s means the algorithm can compress 200 MB of raw FASTQ data every second. Similarly, DS measures the rate at which the original data is restored, capturing the time efficiency of the decompression pipeline during data retrieval.

To avoid ambiguity in storage units, we use decimal units for file-size-derived throughput and cloud-storage examples, and binary IEC units for memory and chunk-size quantities. Specifically, $1\;MB=10^{6}$ bytes and $1\;GB=10^{9}$ bytes are used for compression/decompression speed calculations and storage-cost illustrations. Therefore, CS and DS are computed from the exact input file size in bytes divided by $10^{6}$ and by the elapsed time. In contrast, chunk sizes and peak resident set size (RSS) are reported using binary units: $1\;KiB=2^{10}$ bytes, $1\;MiB=2^{20}$ bytes and $1\;GiB=2^{30}$ bytes. The default FastqCA chunk size is thus 128 MiB , i.e., 134,217,728 bytes.

#### 3.2.2 Assembly quality metrics

To gauge how well an assembly of decompressed reads represents the target genome, we track six complementary statistics.

##### 3.2.2.1 Total length (TL)

The Total Length sums all contig lengths and serves as a proxy for coverage completeness calculated as Equation 4.

$$TL\;=\;\overset{m}{\underset{i=1}{\sum }}\ell_{i}\left(\text{bp}\right).$$ (4)

The closer TL is to the expected genome size, the more sequence has been recovered.

##### 3.2.2.2 N50 and L50

Let the contig lengths be $\left\{\ell_{1},\ldots ,\ell_{m}\right\}$ and sort them in nonincreasing order $\ell_{\left(1\right)}\geq \ell_{\left(2\right)}\geq \cdots \geq \ell_{\left(m\right)}$ . Let $TL=\sum_{i=1}^{m}\ell_{i}$ denote the total assembly length. Assuming k* is defined as Equation 5:

$$k^{*}\;\;=\;\;\min\;\left\{\;k\in \left\{1,\ldots ,m\right\}\;\;:\;\;\overset{k}{\underset{i=1}{\sum }}\ell_{\left(i\right)}\;\;\geq \;\;\frac{1}{2}\;TL\;\right\}.$$ (5)

Then N50 is calculated as Equation 6

$$N50\;\;=\;\;\ell_{\left(k^{*}\right)}\;\;\left(\text{bp}\right),\;L50\;\;=\;\;k^{*}.$$ (6)

N50 is the smallest contig length whose cumulative span reaches at least 50% of TL; larger values denote assemblies dominated by long stretches of sequence. L50 is the minimum number of those longest contigs required to reach the same 50% threshold; smaller values indicate that long contigs dominate, signalling better continuity.

##### 3.2.2.3 Largest contig

As an additional indicator we record the length of the Largest Contig as shown in Equation 7:

$$Largest\;\;Contig\;\;=\;\;\underset{i}{\max}\ell_{i}\;\left(\text{bp}\right).$$ (7)

Larger values suggest better traversal of extended repetitive or coverage-rich regions.

##### 3.2.2.4 Contigs ≥ 10 kbps

Because very long contigs are often critical downstream, we also count contigs whose length is at least 10 kbps as shown in Equation 8,

$$\text{Contigs}\;\geq \;10\;\text{kbp}\;\;=\;\;\overset{m}{\underset{i=1}{\sum }}1\left\{\ell_{i}\geq 10,000\right\}.$$ (8)

More such contigs imply stronger long-fragment recovery.

##### 3.2.2.5 N’s/100 kbp

Reliability is assessed with N’s/100 kbp, defined as Equation 9:

$$N\text{’ }s/100\;\;kbp\;\;=\;\;\frac{N_{\text{N}}}{TL}\times 100,000,$$ (9)

where $N_{\text{N}}$ is the total count of ambiguous ‘N’ bases across all contigs. Lower values indicate fewer unresolved positions and hence greater sequence fidelity.

### 3.3 Compression experiments

To provide a representative evaluation of FASTQ compression capabilities, we compared FastqCA with the general-purpose gzip baseline and with established FASTQ compressors, including FQZcomp, DSRC2, fastqz, Scalce, Quip, NAF and SPRING (Chandak et al., 2019). SPRING provides a directly relevant reference-free comparison because it supports both lossless compression and lossy compression of quality values. Together, the selected tools provide representative coverage of established FASTQ compression approaches under the evaluation settings used in this study. All performance statements in this section refer to the tools, modes and datasets actually benchmarked. Our primary goal for lossless compression was to pursue the optimal compression ratio rather than the fastest compression time. For lossy compression, we aimed to further maximize this ratio by strategically reducing the precision of quality scores, thereby alleviating the storage burden while minimizing the impact on downstream analyses. Accordingly, gzip was run at its maximum compression level (-9), and FastqCA used the LPAQ8 backend with option 9. For other compressors with different command-line interfaces, the specific modes and options used for the corresponding lossless or lossy comparisons are detailed in Supplementary Table S2.

#### 3.3.1 Compression ratio

Table 2 presents the compression ratio (CR) results for ten diverse FASTQ datasets, with the optimal values for each dataset highlighted in bold. In the lossy compression mode, FastqCA achieves the highest CR on nine of the ten datasets, with values ranging from 13.46 × on SRR2093872_1 to 18.48 × on SRR14139158_1. The only exception is DRR057887_1, on which SPRING attains 16.09 × versus the 14.52 × of FastqCA. On the SRR554369 dataset, FastqCA delivers a CR of 15.19 × , which is more than twice the CR of SPRING (6.94 × ) and Scalce (7.41 × ), and represents an improvement of about 88.7% over FQZcomp (8.05 × ). On SRR14139158_1 and SRR14626645_1, FastqCA reaches 18.48 × and 17.26 × respectively, surpassing specialized tools like fastqz, Scalce and SPRING.

**TABLE 2:** Comparison of compressors in compression ratio.

Dataset	Lossy compression						Lossless compression					
	FQZcomp	DSRC2	fastqz	Scalce	SPRING	FastqCA	gzip	DSRC2	Quip	NAF	SPRING	FastqCA
DRR057887_1	11.44	6.80	9.22	13.54	**16.09**	14.52	4.38	6.80	10.31	5.90	10.12	**11.05**
SRR1210085_1	12.90	6.65	8.46	13.89	12.48	**14.32**	4.39	6.65	10.05	5.97	7.94	**10.79**
SRR14139158_1	15.28	6.31	15.47	13.41	13.71	**18.48**	4.36	6.31	10.42	5.95	**13.71**	11.11
SRR14139158_2	11.06	5.51	11.10	9.87	10.07	**15.73**	3.90	5.51	8.69	4.89	**10.07**	9.21
SRR14626645_1	13.89	6.29	14.39	12.57	12.78	**17.26**	4.36	6.29	9.96	5.98	**12.78**	10.65
SRR14626645_2	10.58	5.56	10.80	9.63	9.80	**15.12**	3.95	5.56	8.43	4.98	**9.80**	9.01
SRR2093871_1	12.51	6.54	8.65	13.74	15.40	**16.57**	4.12	6.54	9.92	5.22	8.95	**10.40**
SRR2093872_1	11.33	6.37	7.85	11.14	11.46	**13.46**	4.04	6.37	9.46	5.19	7.22	**9.97**
SRR29245815	8.65	6.15	–	8.60	11.93	**14.99**	2.92	6.15	7.07	4.03	6.06	**8.53**
SRR554369	8.05	5.42	–	7.41	6.94	**15.19**	2.87	5.42	4.63	3.68	4.84	**5.43**

In the lossless compression mode, FastqCA achieves the highest CR on six of the ten datasets, with values ranging from 5.43 × to 11.11×. It systematically exceeds general-purpose tools like gzip and industry-standard specialized compressors including DSRC2, Quip and NAF. On SRR554369, the CR of FastqCA reaches 5.43 × , surpassing SPRING (4.84 × ), Quip (4.63 × ) and DSRC2 (5.42 × ). On the remaining four datasets (SRR14139158_1, SRR14139158_2, SRR14626645_1 and SRR14626645_2), the CR of SPRING is higher than that of FastqCA, e.g., 13.71 × versus 11.11 × on SRR14139158_1. These four files are the mate files from two Illumina HiSeq 4000 paired-end accessions and belong to the larger paired-end datasets in our benchmark. With respect to read-order handling, SPRING’s short-read compressor uses hash-table searches for prefix/suffix matches to reorder reads internally and stores order information for reconstruction, whereas FastqCA keeps reads in input order throughout and does not include a global read-overlap/reordering stage.

Across the ten datasets, FastqCA records the highest lossy CR in nine cases, whereas SPRING records the highest lossy CR on DRR057887_1. In the lossless setting, FastqCA records the highest CR in six cases, while SPRING records the highest CR on SRR14139158_1, SRR14139158_2, SRR14626645_1 and SRR14626645_2. The corresponding values are listed in Table 2.

The WACR values in Table 3 provide an aggregate view of the same compression-ratio results. When computed from the exact compressed byte sizes, WACR directly measures the compression ratio of the complete benchmark corpus. In the current rounded-CR-based recalculation, FastqCA has the highest ten-dataset WACR in both settings: 15.75 in the lossy setting and 9.95 in the lossless setting. The fastqz WACR is calculated over the eight datasets with valid fastqz results in Table 2; therefore, it is reported only as an eight-dataset summary and is not directly comparable with the ten-dataset WACR values.

**TABLE 3:** Weighted average compression ratio across the benchmark datasets.

Metric	Lossy compression						Lossless compression					
	FQZcomp	DSRC2	fastqz	Scalce	SPRING	FastqCA	gzip	DSRC2	Quip	NAF	SPRING	FastqCA
WACR	12.12	6.10	10.86	11.51	11.68	**15.75**	4.07	6.10	9.22	5.38	9.50	**9.95**

Bold values indicate the highest weighted average compression ratios on the complete benchmark corpus.

The dataset-dependent variation in CR is consistent with FastqCA’s residual coding mechanism. In the nucleotide stream, a correctly predicted cell is stored as the marker value 1, whereas an unmatched cell retains the original mapped symbol; therefore, stronger local similarity among neighbouring reads and positions produces sparser residual matrices and higher CR . The difference between lossy and lossless results is also expected because Q4 quantisation reduces the quality-score alphabet and allows CA prediction on the quality matrix, while the lossless mode preserves the original quality scores and passes them directly to LPAQ8. Thus, variations in local sequence redundancy, quality-score distributions and chunk-level ordering jointly lead to the observed differences in CR across datasets.

#### 3.3.2 Compression/decompression speed

Tables 4, 5 summarize the processing speeds across the evaluated datasets. Across the ten datasets, the three-run mean speeds for FastqCA range from 1.602 to 2.714 MB/s for lossy compression (SD range: 0.003–0.248 MB/s), from 2.080 to 3.385 MB/s for lossless compression (SD range: 0.035–0.171 MB/s), from 1.009 to 1.368 MB/s for lossy decompression (SD range: 0.008–0.020 MB/s), and from 1.530 to 1.996 MB/s for lossless decompression (SD range: 0.020–0.226 MB/s). The sample standard deviation was calculated using the n−1 denominator. The per-dataset mean speeds and standard deviations for FastqCA are provided in Supplementary Table S4.

**TABLE 4:** Comparison of compressors in compression speeds (MB/s).

Dataset	Lossy compression						Lossless compression					
	FQZcomp	DSRC2	fastqz	Scalce	SPRING	FastqCA	gzip	DSRC2	Quip	NAF	SPRING	FastqCA
DRR057887_1	33.69	**195.62**	20.03	88.77	22.24	1.845	12.50	199.55	75.31	**344.03**	19.07	2.482
SRR1210085_1	32.94	**196.85**	16.79	87.74	14.41	2.368	12.26	205.91	72.29	**341.76**	12.81	3.157
SRR14139158_1	32.99	**136.09**	8.66	93.36	13.62	2.232	12.31	148.83	70.85	**339.77**	13.75	3.385
SRR14139158_2	33.17	**140.76**	7.80	76.08	11.26	2.099	10.83	138.96	59.23	**289.55**	11.42	3.263
SRR14626645_1	33.23	**131.63**	12.46	88.08	12.93	2.218	12.08	136.99	69.06	**340.62**	12.07	3.346
SRR14626645_2	33.37	**137.27**	8.84	73.79	11.06	2.290	10.72	136.21	57.44	**292.80**	10.22	3.244
SRR2093871_1	32.36	**133.19**	11.54	72.57	22.15	2.162	10.09	128.38	56.84	**284.54**	17.35	2.732
SRR2093872_1	33.42	**140.21**	8.32	73.06	4.94	2.235	11.35	142.63	58.55	**317.70**	4.18	2.784
SRR29245815	39.85	**140.48**	–	102.77	14.50	2.714	9.85	180.52	76.58	**290.65**	11.12	3.350
SRR554369	39.50	**150.39**	–	88.16	5.41	1.602	11.28	88.30	65.27	**299.82**	4.75	2.080

Compression speeds are measured in decimal MB/s. For FastqCA, values are the mean of three independent runs; the per-dataset standard deviations are provided in Section 3.3 and Supplementary Table S4. For other benchmark tools, values are from single runs. Bold values indicate the highest compression speed.

**TABLE 5:** Comparison of compressors in decompression speeds (MB/s).

Dataset	Lossy decompression						Lossless decompression					
	FQZcomp	DSRC2	fastqz	Scalce	SPRING	FastqCA	gzip	DSRC2	Quip	NAF	SPRING	FastqCA
DRR057887_1	0.73	**362.17**	0.29	12.75	31.01	1.009	6.39	**41.81**	0.37	6.96	29.09	1.530
SRR1210085_1	0.77	**125.84**	2.40	9.49	29.05	1.263	5.86	23.80	3.54	6.46	**26.08**	1.859
SRR14139158_1	0.62	26.77	6.53	9.34	**27.37**	1.149	16.10	**30.12**	24.56	18.08	27.42	1.996
SRR14139158_2	1.03	**26.76**	9.47	18.19	25.17	1.150	13.83	**27.51**	23.32	20.26	19.87	1.869
SRR14626645_1	0.91	**27.86**	8.08	22.88	18.24	1.136	16.40	**25.42**	15.02	20.41	23.23	1.921
SRR14626645_2	1.13	**28.13**	9.34	21.84	21.72	1.135	17.37	**27.67**	17.93	17.72	20.84	1.847
SRR2093871_1	1.38	**27.49**	10.66	26.36	23.44	1.197	**85.93**	33.30	21.62	21.29	25.48	1.762
SRR2093872_1	1.33	24.38	10.89	**24.46**	23.62	1.144	15.80	26.33	**27.43**	21.02	21.77	1.663
SRR29245815	1.53	23.57	–	18.87	**24.95**	1.368	**26.22**	25.76	22.89	17.99	20.74	1.980
SRR554369	2.10	**30.64**	–	12.78	15.14	1.045	**32.29**	23.83	18.37	18.53	14.50	1.559

Decompression speeds are measured in decimal MB/s. For FastqCA, values are the mean of three independent runs; the per-dataset standard deviations are provided in Section 3.3 and Supplementary Table S4. For other benchmark tools, values are from single runs. Bold values indicate the highest decompression speed.

FastqCA shows lower throughput than DSRC2, NAF and SPRING because it performs additional CA-based predictive preprocessing before LPAQ8 coding. This lower throughput mainly arises from FastqCA’s compression-density-oriented preprocessing. Before LPAQ8 coding, FastqCA constructs nucleotide residual matrices through CA-based prediction; in the lossy mode, the same predictive step is also applied to the Q4 quality matrix. These additional modeling steps increase computation but contribute to the compression-ratio gains observed in Tables 2. The dataset-to-dataset speed variation is also expected because runtime depends not only on input byte size, but also on read length, read count, identifier structure, quality-score distribution and chunk-level workload balance.

**TABLE 6:** Comparison of compressors in peak memory usage (MiB).

Dataset	Lossy compression						Lossless compression					
	FQZcomp	DSRC2	fastqz	Scalce	SPRING	FastqCA	gzip	DSRC2	Quip	NAF	SPRING	FastqCA
DRR057887_1	1299.62	299.46	1285.69	1472.60	**296.03**	7838.60	**1.69**	403.61	392.25	15.20	296.40	6224.21
SRR1210085_1	1333.17	993.67	1527.00	5247.50	**334.81**	14762.29	**1.88**	1064.94	392.81	15.60	336.86	8434.90
SRR14139158_1	1333.30	1025.53	1459.39	5231.40	**329.02**	11611.24	**1.88**	1029.50	382.50	16.90	329.22	5803.51
SRR14139158_2	1333.00	1072.66	1459.40	5241.50	**329.46**	12012.89	**1.69**	1073.21	383.25	15.80	329.26	5837.03
SRR14626645_1	1333.37	1021.43	1459.12	5237.20	**328.94**	11713.00	**1.88**	1020.38	383.25	15.80	329.00	5808.51
SRR14626645_2	1333.00	1063.14	1459.40	5242.80	**329.32**	12015.16	**1.69**	1063.03	383.81	15.40	329.11	5850.85
SRR2093871_1	1327.50	876.96	1356.13	2317.50	**291.13**	10770.59	**1.88**	1056.94	389.81	15.60	291.13	7992.19
SRR2093872_1	1328.26	990.12	1367.55	2385.60	**295.21**	11621.82	**1.69**	1074.25	389.81	15.40	317.06	8006.64
SRR29245815	1330.32	975.58	–	3120.40	**358.64**	10100.78	**1.50**	1091.29	380.06	15.20	345.99	7139.07
SRR554369	1317.31	**235.43**	–	1586.40	433.82	6361.84	**1.50**	311.41	382.50	12.80	455.18	6806.52

Values are peak resident set size in MiB. Bold values indicate the lowest memory usage within each block (lossy/lossless) for a given dataset.

To reduce the computational cost, FastqCA parallelizes independent chunks using four threads. This design is most relevant to storage-oriented or bandwidth-limited workflows, where higher compression density can be preferred over maximum throughput.

#### 3.3.3 Cost–benefit illustration

To quantify the above trade-off in monetary terms, we use the largest dataset in our benchmark, SRR1210085_1, as an example. This file is 15.1 GB before compression (Table 1). With the lossy compression ratio of 14.32 reported in Table 2, FastqCA reduces the file to about 1.05 GB, saving about 14 GB of storage per file. The compression time is about 1.8 h on the experimental platform. Using public AWS US East prices as an illustrative reference, S3 Standard storage costs 0.023 USD per GB-month, internet egress costs 0.09 USD per GB, and an on-demand r6i.2xlarge instance with 8 vCPUs and 64 GiB RAM costs 0.504 USD per hour. Under these prices, the one-time compute cost is about 0.90 USD per file, whereas the storage saving is about 0.32 USD per file per month. For 1,000 similar samples, the one-time compute cost is about 900 USD, the annual storage saving is about 3,880 USD, and the 5-year net storage saving is about 18,500 USD before any bandwidth saving is counted. These values are intended as an illustrative cost calculation based on public cloud prices.

#### 3.3.4 Peak memory usage

Tables 6, 7 report the peak resident set size (RSS) for compression and decompression, respectively, in MiB. Table 6 shows that FastqCA requires substantially more memory than the other compressors in the benchmark, with total RSS of approximately 6.2–14.4 GiB in lossy mode and 5.7–8.2 GiB in lossless mode. Table 8 shows total decompression RSS of approximately 3.5–6.9 GiB in lossy mode and 2.7–4.8 GiB in lossless mode. Local-context tools such as DSRC2, gzip and NAF use orders of magnitude less memory, and the reference-free compressor SPRING peaks at 291–455 MiB during compression and 257–467 MiB during decompression with a single thread. The higher footprint follows from the chunk-level representation used by the cellular-automaton model, where reads are parsed into in-memory records and transformed into integer matrices before back-end coding. The lossy mode is generally more memory demanding because both nucleotide data and Q4-quantized quality scores are encoded through CA residual matrices, whereas the lossless quality stream bypasses this CA prediction step. Under the four-thread configuration (--threads 4) used in the compression benchmark, the compression RSS values correspond to approximately 1.6–3.6 GiB per configured thread in lossy mode and 1.4–2.1 GiB per configured thread in lossless mode.

**TABLE 7:** Comparison of compressors in peak memory usage during decompression (MiB).

Dataset	Lossy decompression						Lossless decompression					
	FQZcomp	DSRC2	fastqz	Scalce	SPRING	FastqCA	gzip	DSRC2	Quip	NAF	SPRING	FastqCA
DRR057887_1	1316.07	448.00	1298.06	1031.60	**263.34**	6014.20	**1.50**	146.27	391.69	218.32	262.82	3090.62
SRR1210085_1	1330.84	1037.43	1526.62	1033.50	**279.71**	6496.09	**1.69**	1179.95	393.38	5750.84	279.61	4935.95
SRR14139158_1	1330.76	365.20	1458.52	1034.20	**353.41**	5012.97	**1.50**	1070.04	382.29	2620.71	353.83	2765.30
SRR14139158_2	1331.41	1137.48	1458.40	1034.40	**360.48**	7048.28	**1.31**	1133.10	383.62	2632.14	360.48	3601.01
SRR14626645_1	1329.84	1056.69	1458.80	1034.60	**359.46**	5136.15	**1.50**	1054.51	382.54	2119.58	359.48	3183.32
SRR14626645_2	1330.53	1113.30	1458.70	1035.80	**363.38**	6077.62	**1.50**	1105.23	382.52	2128.45	363.22	3411.72
SRR2093871_1	1323.47	**148.73**	1370.21	1033.30	256.99	6154.89	**1.69**	327.04	389.86	651.84	257.23	3280.89
SRR2093872_1	1324.26	**232.07**	1382.32	1032.80	270.94	6193.76	**1.69**	257.62	389.81	675.57	271.27	3317.46
SRR29245815	1323.06	447.59	–	1036.10	**354.20**	5282.47	**1.69**	273.45	379.12	965.52	373.95	3613.93
SRR554369	1315.96	**284.92**	–	1034.80	447.97	3598.56	**1.50**	175.68	381.75	169.67	467.04	2994.84

Values are peak resident set size in MiB. Bold values indicate the lowest memory usage within each block (lossy/lossless) for a given dataset.

**TABLE 8:** Comparative analysis of assembly statistics across 10 datasets (lossy mode).

Dataset	Tool	N50 (bp)	L50	Largest contig	N’s/100k	Total length	Ctgs ≥ 10k
DRR057887_1	Scalce	**81329**	**20**	299412	0.04	5252761	**84**
	FastqCA	**81329**	**20**	**299413**	**0.00**	5251209	83
	FQZcomp	**81329**	**20**	299412	**0.00**	**5252762**	**84**
	DSRC2	**81329**	**20**	**299413**	**0.00**	5251361	83
	Original	81329	20	299412	0.00	5252762	84
SRR1210085_1	Scalce	2565	3276	16716	0.33	28271791	65
	FastqCA	3042	2824	17470	**0.01**	28712227	111
	FQZcomp	**3456**	**2517**	**25608**	**0.01**	**28917538**	**152**
	DSRC2	3384	2571	21910	**0.01**	28854826	143
	Original	3491	2492	25609	0.01	28852320	155
SRR14139158_1	Scalce	**1624**	**6191**	**9677**	0.05	28177662	**0**
	FastqCA	1600	6347	8739	**0.04**	**28499669**	**0**
	FQZcomp	1623	6195	9676	0.05	28182633	**0**
	DSRC2	1623	**6191**	9676	0.05	28172621	**0**
	Original	1624	6191	9676	0.05	28177285	0
SRR14139158_2	Scalce	1578	6418	**8739**	**0.01**	**28326905**	**0**
	FastqCA	1535	6492	6385	0.02	27846002	**0**
	FQZcomp	1567	6452	**8739**	**0.01**	28255146	**0**
	DSRC2	**1597**	**6358**	**8739**	**0.01**	28260957	**0**
	Original	1578	6418	8739	0.01	28326158	0
SRR14626645_1	Scalce	1287	**9580**	**12114**	**0.01**	35774562	**6**
	FastqCA	1252	9959	11869	0.02	**36060335**	5
	FQZcomp	**1288**	**9580**	12113	**0.01**	35774349	**6**
	DSRC2	1275	9859	11868	0.02	**36283117**	5
	Original	1287	9583	12113	0.01	35772338	6
SRR14626645_2	Scalce	1207	10590	**16291**	0.02	36909577	**2**
	FastqCA	**1262**	**9902**	**16291**	0.02	36132702	**2**
	FQZcomp	1208	10599	**16291**	**0.01**	**36936462**	**2**
	DSRC2	1224	10267	**16291**	0.02	36356915	**2**
	Original	1276	9692	16291	0.03	35808399	2
SRR2093871_1	Scalce	**103503**	**15**	**336625**	0.05	6243855	**82**
	FastqCA	**103503**	**15**	**336625**	**0.00**	**6245170**	81
	FQZcomp	**103503**	**15**	**336625**	**0.00**	6243717	**82**
	DSRC2	**103503**	**15**	**336625**	**0.00**	**6253264**	81
	Original	103503	15	336625	0.00	6243716	82
SRR2093872_1	Scalce	**100532**	**15**	**336625**	**0.05**	6233444	**85**
	FastqCA	**100532**	**15**	**336625**	0.06	6232152	**85**
	FQZcomp	**100532**	**15**	**336625**	0.10	**6233452**	**85**
	DSRC2	**100532**	**15**	**336625**	**0.05**	6232228	**85**
	Original	100532	15	336625	0.06	6233415	85
SRR29245815	Scalce	647209	**3**	1108976	11.45	4384988	17
	FastqCA	**795604**	**3**	**1363644**	1.58	**5386503**	16
	FQZcomp	781596	**3**	1343922	0.96	5294491	**18**
	DSRC2	784867	**3**	1347505	**0.94**	5311820	**18**
	Original	778698	3	1342317	1.04	5288423	16
SRR554369	Scalce	2687	488	20712	22.20	4837957	19
	FastqCA	3827	363	26167	**0.06**	5235210	64
	FQZcomp	3468	387	33417	0.08	5172706	51
	DSRC2	**4416**	**315**	**36657**	**0.06**	**5250236**	**76**
	Original	3468	387	33417	0.04	5171514	51

For the default 128 MiB chunk size, the main per-thread memory components are the parsed Bio.SeqIO records of the chunk (about 0.4–0.8 GiB for short-read inputs with many records), the integer matrices and image buffers used by the CA transform (about 0.2–0.25 GiB in total), the LPAQ8 back-end process under option 9 (about 1.5–1.6 GiB), and Python interpreter or buffering overhead (about 0.1 GiB). The record and matrix components scale approximately with the chunk size and the number of reads in the chunk, whereas the LPAQ8 component is incurred per active thread. Therefore, users can reduce peak RSS by lowering --threads or --block_size; the former reduces the number of concurrent per-thread working sets, and the latter reduces the chunk-dependent record and matrix terms, at a moderate cost in throughput and sometimes compression ratio.

### 3.4 Assembly experiments

To assess the impact of lossy compression on genome assembly, we benchmarked FastqCA, Scalce, FQZcomp, and DSRC2 using a standard workflow (SPAdes → minimap2 → Racon → QUAST) against uncompressed references. Notably, fastqz was excluded from this study as its decompressed output violated FASTQ format standards, causing fatal errors in the polishing stage (Racon).

Our evaluation encompasses ten heterogeneous datasets (as detailed in Table 8), ranging from DRR057887_1 to SRR554369. These datasets were chosen to represent a broad spectrum of genomic characteristics, sequencing depths, and quality distributions, ensuring that the assessment of assembly fidelity is robust across varying data complexities.

**TABLE 9:** Ablation study on the CA module of FastqCA.

Dataset	CR in lossless mode				CR in lossy mode		
	Full	w/o CA	w/o mapping	Gain (%)	Full	w/o CA	Gain (%)
SRR14139158_1	**11.11**	10.16	10.10	9.37	**18.48**	15.98	15.66
SRR14626645_1	**10.65**	9.80	9.62	8.62	**17.26**	15.14	14.02
SRR14139158_2	**8.71**	8.11	7.87	7.41	**15.73**	13.86	13.42
SRR14626645_2	**8.60**	8.04	7.75	6.97	**15.12**	13.46	12.27
SRR554369	**5.43**	4.91	5.03	10.57	**15.19**	11.69	29.95
DRR057887_1	**7.53**	7.20	7.01	4.56	**14.52**	13.33	8.93
SRR1210085_1	**6.67**	6.39	6.23	4.50	**14.32**	13.03	9.90
SRR2093871_1	**7.21**	6.64	6.63	8.71	**16.57**	13.79	20.16
SRR2093872_1	**6.24**	6.10	5.88	2.30	**13.46**	12.80	5.10
SRR29245815	**5.57**	5.10	5.27	9.25	**14.99**	12.01	**24.82**
Average	–	–	–	8.12	–	–	16.58

“Full” denotes the proposed FastqCA method; “w/o CA” denotes the baseline variant without the Cellular Automaton predictive module; “w/o mapping” denotes the lossless variant without the integer mapping stage. “Gain” represents the relative improvement in compression ratio, calculated as $\left({\text{CR}}_{\text{Full}}-{\text{CR}}_{\text{w/o CA}}\right)/{\text{CR}}_{\text{w/o CA}}\times 100\%$ . The highest values and significant gains are highlighted in bold.

Table 8 presents the detailed assembly statistics, where the superior performance among the lossy compressors (Scalce, FastqCA, FQZcomp, and DSRC2) is highlighted in bold for each metric. Notably, for high quality score datasets such as DRR057887_1 and SRR2093871_1, the original assemblies contain negligible ambiguous bases. FastqCA and FQZcomp successfully maintain this structural fidelity (yielding 0.00 N’s/100 kbp), whereas Scalce introduces minor assembly errors (0.04–0.05 N’s/100 kbp). On datasets SRR14139158_1 and SRR14139158_2, zero counts were observed for contigs ≥ 10 kbp across all evaluated tools, This uniform outcome suggests that the fragmentation is attributed to the inherent characteristics of these specific samples (e.g., limited sequencing depth or short read length) rather than artifacts introduced by the compression algorithms.

#### 3.4.1 Continuity (N50 and L50)

For high quality score datasets such as DRR057887_1 and SRR2093871_1, the N50 and L50 values produced by FastqCA are identical or nearly identical to those of the uncompressed original baseline (e.g., maintaining an N50 of 81,329 bp and 103,503 bp, respectively). This indicates that the Q4 quantization strategy preserves the local topology of long fragments without disrupting assembly connectivity. Table 8 reports the assembly statistics obtained from the original FASTQ files and from each lossy-compressed input, including FastqCA-decompressed reads. Prior studies provide context for the use of reduced-resolution quality scores in downstream analyses. Yu et al. (2015) reported that SNP-calling accuracy can be improved by discarding 95% of quality scores. Ochoa et al. (2017) systematically evaluated several lossy quality-compression schemes and reported variant-calling performance that, under some configurations, is superior to that obtained from uncompressed data. Most directly relevant to our setting, Rivara-Espasandín et al. (2022) applied a four-level quality quantizer to nanopore data from a mock microbial community and observed fewer mismatches per 100 kbp after *de novo* assembly polishing than with the non-quantised data. These studies provide a precedent for evaluating reduced-resolution quality scores through downstream task metrics. In our experiments, the Q4 mode retains assembly-level statistics comparable to the original FASTQ files across the evaluated datasets, with the dataset-wise values reported in Table 8.

#### 3.4.2 Global integrity (total length and contigs ≥10 kbp)

For FastqCA, deviations in total assembly length are minimal (typically within 2% ) across all evaluated datasets. Specifically, on the SRR554369 dataset, the total length is 1.23% above the uncompressed baseline. The count of contigs ( ≥10 kbp) on SRR554369 is 64 for FastqCA versus 51 for the uncompressed baseline; the per-tool counts on the other nine datasets are reported in Table 8.

#### 3.4.3 Local resolvability and gaps (largest contig and Ns/100 kbp)

Regarding local resolvability, on the large-scale dataset SRR29245815 the largest contig produced by FastqCA is 1,363,644 bp, compared with 1,342,317 bp on the uncompressed baseline. In terms of gaps, FastqCA exhibits a marginal increase in the ambiguous base rate on SRR554369 (from 0.04 to 0.06 Ns/100 kbp); for reference, recent high-quality chromosome-level assemblies report gap densities around 0.28 Ns/100 kbp and consider this a substantial achievement (Chen et al., 2025).

On the literature-linked *P. aeruginosa* benchmark dataset SRR554369, the assembly obtained from FastqCA-decompressed reads has a total length and ambiguous-base rate close to the uncompressed baseline. The corresponding N50, L50, largest-contig and contig-count values are reported in Table 8, together with the original FASTQ file and the other lossy-compressed inputs. Together with the compression ratios above, these results indicate that the Q4 mode reduces storage while retaining assembly-level statistics comparable to the original FASTQ file in this benchmark.

### 3.5 Ablation study

To quantify the individual contribution of the cellular automaton (CA) based predictive modeling to the overall compression performance, we conducted an ablation study. We implemented a baseline variant of FastqCA by removing the CA prediction module. In this baseline configuration, the integer-mapped matrices (Matrix G for sequences and Matrix Q for quality scores) were fed directly into the backend LPAQ8 compressor without the intermediate predictive transformation. We evaluated both the full FastqCA method and the baseline variant across 10 representative datasets in both lossless and lossy modes.

The comparative results are presented in Table 9. The experimental data demonstrates that the CA module is critical for achieving high compression ratios (CR). In the lossless mode, the inclusion of the CA module resulted in an average CR improvement of approximately 8.12%. Notably, on the SRR554369 dataset, the lossless compression ratio increased by 10.57% (from 4.91 × to 5.43 × ). As shown by the “w/o mapping” column in Table 9, removing only the integer mapping yields a lossless CR comparable to the “w/o CA” baseline, indicating that the integer mapping and the CA predictive module act as successive stages of a single predictive-modeling pipeline, and the reported lossless gain reflects the contribution of this pipeline as a whole.

The contribution of the CA module is even more pronounced in the lossy compression mode, yielding an average CR gain of 16.58%. For datasets with high local redundancy, such as SRR554369 and SRR29245815, the full FastqCA algorithm achieved gains of 29.95% and 24.82%, respectively, compared to the baseline. This significant enhancement verifies that the CA-based predictive modeling effectively captures two-dimensional spatial correlations across reads. By converting the original dense integer matrices into highly sparse difference matrices ( $G_{\mathrm{prime}}$ and $Q_{\mathrm{prime}}$ ), the CA module substantially reduces the conditional entropy of the data stream, thereby maximizing the compression performance of the backend context-mixing algorithm.

Although the predictive modeling step introduces additional computational overhead, the substantial reduction in storage size justifies its application, particularly in large-scale genomic data archiving scenarios where storage efficiency is paramount.

## 4 Conclusion

The compression of high-throughput sequencing data remains a critical challenge. We proposed FastqCA, a reference-free, cellular-automaton-based compressor that exploits two-dimensional spatial redundancy. FastqCA supports a lossless mode with competitive performance and a lossy mode that enhances efficiency through optimized quantization. Experiments on ten heterogeneous datasets confirm that FastqCA preserves genome assembly continuity. On the complex SRR554369 dataset, the assembly obtained from FastqCA-decompressed reads has an N50 of 3,827 bp, 64 contigs ≥ 10 kbp and an ambiguous-base rate of 0.06 N’s/100 kbp; the per-tool values are reported in Table 8.

Several practical considerations should be noted. The current Python implementation and CA-based preprocessing introduce additional runtime and memory overhead, so FastqCA is more suitable for storage-oriented or bandwidth-limited workflows in large-scale genome-analysis settings than for speed-first compression scenarios (Patil et al., 2024). In addition, the lossy Q4 mode is not bitwise reversible for quality scores; therefore, the lossless mode should be used when exact quality preservation is required, and additional validation is needed before applying the lossy mode to downstream analyses beyond the assembly metrics evaluated here. Future work will focus on improving execution efficiency, reducing temporary-file overhead and extending evaluation to broader sequencing data types and downstream tasks, consistent with the continuing need for scalable genome-analysis infrastructure (Al-Aamri et al., 2023).

## Data Availability

The original datasets are publicly available in the NCBI repository: https://www.ncbi.nlm.nih.gov/, the accession numbers are included in the article. The original contributions presented in the study are publicly available. This data can be found here: https://github.com/XXhaos/FastqCA.

## Footnotes

1
https://mattmahoney.net/dc/lpaq8.zip
