# Supplementary material for: FastqCA: an effective FASTQ compressor through 2D spatial redundancy reduction
Source: Front Genet. 2026 Aug 14;17:1827471. doi: 10.3389/fgene.2026.1827471 (PMC13521550; doi:10.3389/fgene.2026.1827471)
Supplement: Supplementary file 1 [file DataSheet1.pdf]

FastqCA: An Effective FASTQ Compressor through 2D Spatial Redundancy Reduction

Supplementary File

Supplementary Table 1: Sensitivity of FastqCA's compression ratio in lossless mode.

Dataset	Compression ratio				Compression speed (MB/s)				Peak memory (MiB)			
	16 MiB	32 MiB	64 MiB	128 MiB	16 MiB	32 MiB	64 MiB	128 MiB	16 MiB	32 MiB	64 MiB	128 MiB
DRR057887_1	6.270	6.610	7.070	11.050	2.030	1.880	1.560	3.610	5353.950	5765.060	5353.230	6224.210
SRR1210085_1	6.110	6.310	6.470	10.790	1.750	1.780	1.770	3.620	5495.830	5901.520	6773.510	8434.900
SRR14139158_1	9.150	9.740	10.400	11.110	1.990	2.080	2.050	3.520	4447.780	4601.320	4491.520	5803.510
SRR14139158_2	7.520	7.890	8.290	9.210	1.980	1.990	2.040	3.100	4520.950	4291.730	5073.060	5837.030
SRR14626645_1	8.940	9.450	10.030	10.650	2.170	2.350	2.390	3.450	4415.860	4678.570	5066.860	5808.510
SRR14626645_2	7.500	7.840	8.210	9.010	1.970	2.040	2.130	3.020	4520.440	4723.770	4652.760	5850.850
SRR2093871_1	6.410	6.700	7.030	10.400	2.080	2.070	2.020	2.520	4430.890	5817.930	6530.570	7992.190
SRR2093872_1	5.740	5.940	6.160	9.970	1.920	2.060	1.920	3.080	5436.670	5834.130	6488.000	8006.640
SRR29245815	4.850	5.070	5.340	8.530	2.670	2.330	2.280	2.000	4967.400	5746.480	6223.700	7139.070
SRR554369	4.530	4.760	5.090	5.430	2.990	2.040	1.960	2.160	5372.890	5589.690	5199.280	6806.520

Chunk sizes are binary IEC units: $1\,\text{MiB} = 2^{20}$ bytes. Compression speed is reported in decimal MB/s, where $1\,\text{MB} = 10^6$ bytes. Peak memory is reported as MiB.

Supplementary Table 2 : Compressor used in our experiments

Algorithm	Key Options	Version	Website
FQZcomp	-n2 -s7+ -b -q3	4.6	https://github.com/jkbonfield/fqzcomp
DSRC2 (lossless)	c -m2	2.0.2	https://github.com/refresh-bio/DSRC
DSRC2 (lossy)	c -l[b]	2.0.2	https://github.com/refresh-bio/DSRC
fastqz	cQ[a]	1.5	https://mattmahoney.net/dc/fastqz/
gzip	-9	1.10	https://www.gnu.org/software/gzip/
Quip	default mode	1.1.8	https://github.com/dcjones/quip
Scalce (lossy)	-p 30[c]		https://github.com/sfu-compbio/scalce
NAF	--temp-dir <tmp>	1.3.0	https://github.com/KirillKryukov/naf
SPRING (lossless)	-c -t 1 -w <tmp>	1.1.0	https://github.com/shubhamchandak94/SPRING
SPRING (lossy)	-c -t 1 -w <tmp> -q ill_bin	1.1.0	https://github.com/shubhamchandak94/SPRING
FastqCA (lossy)	--compressor Lossy --mode c --threads 4		https://github.com/XXhaos/FastqCA
FastqCA (lossless)	--compressor Lossless --mode c --threads 4		https://github.com/XXhaos/FastqCA

gzip was run with its maximum compression level, -9. FastqCA used four workers and LPAQ8 backend at option 9.
[a] fastqz uses Q as quality quantization parameter in cQ.
[b] DSRC lossy uses -l flag.
[c] SCALCE lossy option -p 30.

Supplementary Table 3 : FASTQ datasets used in our experiments

Dataset Name	SRA Link
DRR057887_1	`https://www.ncbi.nlm.nih.gov/sra/?term=DRR057887`
SRR1210085_1	`https://www.ncbi.nlm.nih.gov/sra/?term=SRR1210085`
SRR14139158_1	`https://www.ncbi.nlm.nih.gov/sra/?term=SRR14139158`
SRR14139158_2	`https://www.ncbi.nlm.nih.gov/sra/?term=SRR14139158`
SRR14626645_1	`https://www.ncbi.nlm.nih.gov/sra/?term=SRR14626645`
SRR14626645_2	`https://www.ncbi.nlm.nih.gov/sra/?term=SRR14626645`
SRR2093871_1	`https://www.ncbi.nlm.nih.gov/sra/?term=SRR2093871`
SRR2093872_1	`https://www.ncbi.nlm.nih.gov/sra/?term=SRR2093872`
SRR29245815	`https://www.ncbi.nlm.nih.gov/sra/?term=SRR29245815`
SRR554369	`https://www.ncbi.nlm.nih.gov/sra/?term=SRR554369`

Supplementary Table 4: Per-dataset compression and decompression speeds for FastqCA across all ten datasets (mean ± standard deviation)

Dataset	**Compression Speed (MB/s)**		**Decompression Speed (MB/s)**	
	FastqCA (lossy)	FastqCA (lossless)	FastqCA (lossy)	FastqCA (lossless)
DRR057887_1	1.845 ± 0.015	2.482 ± 0.037	1.009 ± 0.008	1.530 ± 0.020
SRR1210085_1	2.368 ± 0.148	3.157 ± 0.124	1.263 ± 0.020	1.859 ± 0.094
SRR14139158_1	2.232 ± 0.100	3.385 ± 0.117	1.149 ± 0.008	1.996 ± 0.086
SRR14139158_2	2.099 ± 0.248	3.263 ± 0.118	1.150 ± 0.012	1.869 ± 0.207
SRR14626645_1	2.218 ± 0.049	3.346 ± 0.035	1.136 ± 0.020	1.921 ± 0.217
SRR14626645_2	2.290 ± 0.007	3.244 ± 0.111	1.135 ± 0.012	1.847 ± 0.213
SRR2093871_1	2.162 ± 0.003	2.732 ± 0.171	1.197 ± 0.009	1.762 ± 0.184
SRR2093872_1	2.235 ± 0.012	2.784 ± 0.158	1.144 ± 0.012	1.663 ± 0.176
SRR29245815	2.714 ± 0.024	3.350 ± 0.115	1.368 ± 0.008	1.980 ± 0.226
SRR554369	1.602 ± 0.009	2.080 ± 0.083	1.045 ± 0.012	1.559 ± 0.152
